# Metabolic issues in patients affected by schizophrenia: clinical characteristics and medical management

**DOI:** 10.3389/fnins.2015.00297

**Published:** 2015-09-03

**Authors:** Antonio Ventriglio, Alessandro Gentile, Eleonora Stella, Antonello Bellomo

**Affiliations:** Department of Clinical and Experimental Medicine, University of FoggiaFoggia, Italy

**Keywords:** diabetes, weight gain, metabolic syndrome, schizophrenia, psychosis, antipsychotics agents, guidelines

## Abstract

Patients affected by psychotic disorders are more likely to develop high rates of co-morbidities, such as obesity, type 2 diabetes, dyslipidemias, hypertension, metabolic syndrome, myocardial infarction, stroke etc., in the long-term. These morbidities have a significant impact on the life-expectancy of these patients. Patients with chronic psychoses show a 2–3-fold increased risk of death mostly from cardiovascular and metabolic diseases. Although there may be an independent link, between schizophrenia and metabolic conditions the cardio-metabolic risk is mostly related to an unhealthy lifestyle and the usage of antipsychotic agents (especially Second Generation Antipsychotics or atypical) even when these remain effective treatments in the management of major psychoses. Recently, many international organizations have developed screening and monitoring guidelines for the control of modifiable risk factors in order to reduce the rate of co-morbidity and mortality among patients affected by schizophrenia. This paper is a review of current knowledge about the metabolic issues of patients affected by schizophrenia and describes clinical characteristics and medical management strategies for such conditions.

## Introduction

Patients diagnosed with psychotic disorders report a reduced expectancy of life and show higher rate of physical co-morbidities said to be mostly due to an unhealthy life style and as a result of treatment by antipsychotic drugs (Saha et al., 2007; Fleischhacker et al., 2008).

It has been described that patients affected by schizophrenia show a 2–3-fold increased risk of death compared to the general population as a result of suicide and cardiovascular and metabolic diseases (Saha et al., 2007; Fleischhacker et al., 2008). In 2004, the American Diabetes Association (ADA) defined the Cardio-metabolic Risk (CMR) as a cluster of many risk factors (ADA website^1^). Some of them are considered to be modifiable with dietary changes, physical exercise (e.g., overweight/obesity, high blood glucose, hypertension, abnormal lipid metabolism, physical inactivity, smoking) whereas others are seen as non-modifiable (e.g., age, race, ethnicity, gender, family history). The prevalence and relative risk of modifiable factors are not only higher in the patients affected by psychotic conditions but such individuals may also show relatively poor access to health care system and higher levels of unhealthy lifestyle (Druss et al., 2001; Cradock-O'Leary et al., 2002; Newcomer, 2005; Correll, 2007; Fleischhacker et al., 2008).

Antipsychotic agents (APs) have been shown to greatly influence those modifiable risk factors leading to weight gain and metabolic changes among all patients treated (Newcomer, 2007; Nasrallah, 2008; Papanastasiou, 2013). In particular, Metabolic Syndrome (constituting a cluster of clinical conditions such as abdominal obesity, insulin resistance, dyslipidaemias, hypertension) is well known to be highly prevalent among individuals affected by psychosis. Metabolic syndrome is also well recognized as being associated with an increased cardiovascular risk and resulting mortality (Papanastasiou, 2013). Patients diagnosed with schizophrenia have been shown to be overweight/obese (45–55%, RR: 1.5–2), smokers (50–80%, RR: 2–3), to report diabetes (10–15%, RR: 2), hypertension (19–58%, RR: 2–3), dyslipidemias (25–69% RR ≤ 5) and metabolic syndrome (37–63%, RR: 2–3) (Correll, 2007). The median all- causes standardized mortality ratio (SMR) for people affected by schizophrenia has increased over the decades from 1.84 in the 1970s to 3.20 in 1990s (De Hert et al., 2009a). Also, cardiovascular mortality in patients with schizophrenia also increased in those decades especially among men (Osby et al., 2000). As stated above, medical co-morbidities lead to a 20% shorter lifespan among patients diagnosed with psychoses (Harris and Barraclough, 1998; Castillo Sánchez et al., 2014; Ifteni et al., 2014; McLean et al., 2014). In particular, schizophrenia is significantly associated with premature mortality and a higher rate of sudden, unexpected death mostly as a result of cardiovascular, respiratory, and neurological disorders (Ifteni et al., 2014). Early recognition and treatment of such co-morbidities should, therefore, be a priority for clinicians who treat patients with psychoses since the standard risk assessment underestimates the risk of death in schizophrenia (Harris and Barraclough, 1998; Ifteni et al., 2014). Also, evidence from past few decades has confirmed that there may be a link between major mental illness and metabolic conditions without any influence of treatments since metabolic disturbances have been found in drug-naïve patients with first-episode illness (Ryan et al., 2003).

Genetic links between diabetes and schizophrenia were recognized by Henry Maudsley in his 1897 textbook “*The Pathology of Mind*” (Mausdley, 1897). Similarly, weight gain and abnormal eating behaviors were recognized by Kraepelin and Bleuler in the beginning of the twentieth century. These observations have been recently confirmed in recent studies showing that first- episode and drug naïve patients with psychosis report impaired fasting glucose, insulin- resistance and higher levels cortisol than healthy controls (Ryan et al., 2003; Thakore, 2004). They also show higher rates of visceral obesity related to a subtle disturbance of the hypothalamic-pituitary-adrenal axis as alterations of hormonal and immune pathways are recognized in the blood samples of patients with schizophrenia (Thakore, 2004). Van Beveren et al. suggest that the presence of a molecular endophenotype including the disruption of insulin and growth factor also signals pathways as a risk factor for schizophrenia (Phutane et al., 2011; van Beveren et al., 2014). Moreover, drug naïve/first episode schizophrenia patients present increased levels of IL-1β, IL-6, and TNF-α and higher adiponectin levels that may play a pro-inflammatory role leading to later metabolic syndrome (Song et al., 2013).

Obviously, cardio-metabolic risk factors are lower among drug-free/first episode patients than treated schizophrenia patients, and the cardiovascular risk is not significantly higher at this stage of illness. This indicates that first- episode patients should be screened in order to put primary prevention of cardiovascular morbidity and mortality in place (Phutane et al., 2011; Mitchell et al., 2013).

## The role of antipsychotic agents

Psychopharmacological agents are effective and necessary for the management and relapse- prevention of psychotic disorders: untreated psychotic patients show much higher mortality and suicide rates, more hospitalizations and worst outcomes of illness with greater cognitive and functional impairment (Tiihonen et al., 2006; De Hert et al., 2009a).

However, there is a considerable literature on the metabolic adverse effects of antipsychotic agents and, in particular, Second Generation Antipsychotics (SGAs) appear to induce more weight gain and metabolic abnormalities than First Generation Antipsychotics (FGAs) (Newcomer, 2007; Nasrallah, 2008; De Hert et al., 2009a; Papanastasiou, 2013).

Clinicians employing newer antipsychotics deal with metabolic side effects more frequently than extrapyramidal symptoms and tardive dyskinesia. SGAs are responsible of new cases of diabetes, probably due to specific diabetogenic actions, in particular by drugs such as clozapine and olanzapine (Reynolds and Kirk, 2010; Stahl, 2013). The diabetogenic mechanism may involve a muscarinic M3- receptor antagonism (which is perhaps more relevant for olanzapine and clozapine) since M3 receptors are localized on beta- cells of pancreas and regulate the insulin release and glucose levels homeostasis (Stahl, 2013). Also, M3- receptors blockage might alter glucose metabolism leading to diabetes, diabetic ketoacidosis, hyperosmolar syndrome, all observed in patients treated with olanzapine and clozapine (Reynolds and Kirk, 2010). Weight gain and dyslipidemias (in particular hypertriglyceridemia) are also known to be trigger factors for diabetes and are frequently induced by antipsychotics like olanzapine and clozapine, whereas aripiprazole and ziprasidone are associated with some weight- loss or poor weight-gain (Reynolds and Kirk, 2010; Stahl, 2013; Table 1). Weight gain might also be induced by 5HT2C- and H1- receptor affinity of antipsychotic molecules: H1- receptors are localized in the hunger and satiety centers held in the hypothalamus and are responsible for hyperphagia (Reynolds and Kirk, 2010; Stahl, 2013). Other receptor mechanisms may have additive or synergistic effects. For example, dopamine D2 receptor antagonism can enhance 5-HT2C-mediated effects on food intake, as well as influencing lipid and glucose metabolism via disinhibition of prolactin secretion (Reynolds and Kirk, 2010; Stahl, 2013). SGAs also have an impact on the hypothalamic regions involved in the control of food-intake for example, olanzapine increases NPY (neuropeptide Y) expression in the ARC (Arcuate Nucleus) (Reynolds and Kirk, 2010; Stahl, 2013).

**Table 1:** **Side effects of SGAs (adapted from American Diabetes Association/American Psychiatric Association, 2004; De Nayer et al., 2005; Cohn and Sernyak, 2006; De Hert et al., 2006; Tschoner et al., 2007; Leucht et al., 2009b)**.

	**Weight gain**	**Dyslipidemia**	**Diabetes**	**EPS**	**Prolactin increase**	**Sedation**	**QT- interval prolongation**
Amisulpride	+	−	+	+	+++	−	−
Aripiprazole	−	−	−	+/−	−	+	−
Asenapine	+/−	+/−	−	−	−	++	−
Clozapine	+++	++	+++	−	−	+++	+
Olanzapine	+++	++	+++	−	−	+++	−
Quetiapine	++	+	++	−	−	++	−
Risperidone	++	+/−	++	+	+++	+	++
Ziprasidone	+/−	−	−	−	+	−	++

*+++, high incidence;++, moderate incidence, +, low incidence, −, very low incidence; EPS, extra-pyramidal symptoms*.

Between 15 and 72% of patients affected by psychotic disorders report a significant weight gain during the acute and maintenance treatment of their illness (De Hert et al., 2009a). Studies have shown a significant 10-weeks weight gain during the antipsychotic treatment which ranges widely with different medications. For example, this weight gain for clozapine is around (+4.45 kg) higher than that gained by olanzapine (+4.15 kg) which is higher than quetiapine and risperidone (+2.1 kg) which in turn are higher than those occurring as result of treatment with aripiprazole and ziprasidone (< 1 kg) (Allison et al., 1999; De Hert et al., 2009a). These findings have been confirmed by clinical trials such as CATIE (Clinical Antipsychotic Trials of Intervention Effectiveness) and EUFEST (European First Episode Schizophrenia Trial) Studies (Goff et al., 2005; Lieberman et al., 2005; Daumit et al., 2008; De Hert et al., 2009a; Leucht et al., 2009a).

Clinical factors associated with higher weight gain are younger age, lower initial BMI, personal and family history of obesity, non-white ethnic background, tendency to overeat in time of stress, cannabis use and first episode psychosis (De Hert et al., 2009a). Weight gain is not related to SGAs doses used, except for olanzapine and clozapine (Simon et al., 2009).

There are some putative genes involved in the drug- induced weight gain: the most replicated findings are regarding the genes for melanocortin 4 receptor (MC4R), serotonin 2C receptor (HTR2C), leptin, neuropeptide Y (NPY) and cannabinoid receptor 1 (CNR1) (Shams and Müller, 2014).

Causality between antipsychotic usage and the onset of diabetes can be difficult and as noted earlier, people with psychotic disorders tend to show an increased risk for diabetes even when drug- naïve or untreated (De Hert et al., 2009a).

Approximately 15% of patients with psychosis go on to develop type 2 diabetes (Bushe and Holt, 2004). Since the prevalence of diabetes among schizophrenia patients is higher than in the general population (2-fold increased risk), in 2008 the Canadian Diabetes Association (CDA) defined schizophrenia as an independent risk factor for type 2 diabetes including the usage of second generation antipsychotics in the list of risk factors (Canadian Diabetes Association Clinical Practice Guidelines Expert Committee et al., 2013).

In addition, clozapine and olanzapine might influence insulin secretion with a direct stimulating effect on pancreatic β-cells (Melkersson et al., 2004). A significantly higher increase of HbA1c levels with: olanzapine (0.4%) > quetiapine (0.04%) > risperidone (0.07%) > perphenazine (0.09%) > ziprasidone (0.11%) has been demonstrated thereby suggesting that not all drugs are the same (Lieberman et al., 2005).

According to the evidence, a baseline assessment of fasting- glucose is relevant in order to screen new cases of diabetes developing as a result of antipsychotic treatments. Clinicians managing patients affected by chronic psychoses need to make better and more focused efforts to prevent diabetes and respond to it early when it does occur (Ventriglio et al., 2014).

Coronary Artery Disease (CAD) includes the increase of low-density lipoprotein cholesterol (LDL-C) and/or decrease of high-density lipoprotein cholesterol (HDL-C). Studies have shown that the usage of antipsychotic drugs is associated with an increase of LDL-C and a decrease of HDL-C (Goff et al., 2005; Daumit et al., 2008). The increase of LDL cholesterol may differ depending on which anti-psychotic drugs are used. As seen before, clozapine and olanzapine are two compounds which are associated with the higher increase LDL and triglycerides in serum (Goff et al., 2005; Daumit et al., 2008). It is important that the clinicians respond rapidly in the early phase of treatment when weight gain is most likely to occur (Goff et al., 2005; Daumit et al., 2008). It has been recommended that clinicians should measure lipid levels every 2–3 months following the commencement of treatment and also once weight- gain has reached stable levels (De Hert et al., 2009a). Lipid concentration can be reduced rapidly following a psycho—educational and monitoring program focused on diet, physical exercise and better life- style (Ventriglio et al., 2014).

Increase in levels of blood pressure may occur sporadically, mostly during the clozapine treatment (Gardner and Teehan, 2011). In contrast, reduction of blood pressure with associated symptoms of feeling weak is a common occurrence with many antipsychotic treatments. Syncopal episodes related to orthostasis may happen, mostly due to low-potency first-generation antipsychotics (α1-Adrenergic and calcium—receptors blockage is generally involved in this adverse effect). Posture-related drops in blood pressure are especially common with first-generation antipsychotics. A slower dosage titration has been recommended and with most drugs, tolerance develops with continued use, and these effects appear to recede (Gardner and Teehan, 2011).

## Metabolic syndrome and psychosis

The Metabolic Syndrome (MetS; also known as Syndrome X or Reaven's syndrome) constitutes of a cluster of different clinical conditions, including abdominal obesity, insulin resistance, dyslipidaemias, and elevated blood pressure. All components of the MetS have been recognized as independent risk factors for cardiovascular disease, pro-thrombotic state, pro-inflammatory state, non-alcoholic fatty liver disease and reproductive disorders (Papanastasiou, 2013).

Criteria for definition of MetS have developed over the decades as more information has emerged. World Health Organization (WHO) produced the definitions in 1985, European Group for the Study of Insulin Resistance in 1999, National Cholesterol Education Program's Adult Treatment Panel III (NCEP-ATP III) in 2001 (modified in 2003), American Association of Clinical Endocrinologists in 2003 and finally by the International Diabetes Federation (IDF) in 2006 (Papanastasiou, 2013).

MetS is diagnosed when 3 out of the following 5 criteria are recognized in the same patients:

1. Increased waist circumference, M > 102 cm/F > 88 cm (IDF: M ≥ 94 cm/F ≥ 80 cm);
2. Elevated triglycerides (≥150 mg/dl) or drug treatment for dyslipidaemia;
3. Reduced high-density lipoprotein (HDL) (< 40 mg/dl) or being on drug treatment for hypercholesterolaemia;
4. Elevated blood pressure (systolic ≥ 130 mmHg, diastolic ≥ 85 mmHg) or a history of hypertension or drug treatment for hypertension;
5. Elevated levels of fasting glucose (≥100 mg/dl) or being on medication treatment for hyperglycaemia.

Papanastasiou (2013) observed that the prevalence rates of MetS vary across samples reported depending upon the variety of patients, their age, sex, ethnicity, medication status, smoking, duration, and outcomes of illness. Generally, the prevalence rates of MetS vary from 3.9 to 68%, including patients treated with FGAs and/or SGAs. It has been noted that of the prevalence of MetS peaks in the fourth/fifth decade of life. Again, clozapine and olanzapine appeared to be related to higher rates of MetS compared with other antipsychotic agents (Centorrino et al., 2012). Also, the comparisons between previously unmedicated and currently treated patients show an increase in prevalence from 24.7 to 49.6% of MetS. Not surprisingly, patients treated with SGAs show much higher rates of MetS compared to those treated with FGAs (27.8 vs. 9.8%) (De Hert et al., 2009b; Papanastasiou, 2013).

Recently it has been reported that MetS may influence the cognitive functioning in patients with chronic psychosis–especially in immediate memory, delayed memory, and attention (Li et al., 2014).

## Prolactin levels

Hyperprolactinemia induced by antipsychotic medication is mostly due to D2-receptors blockade in the tubero-infundibular dopaminergic system of the hypothalamus. The magnitude of hyperprolactinemia correlates strongly with dopamine D2 receptor blockade- potency of the medication being taken. Prolactin levels may produce clinical signs and consequences: galactorrhea, gynecomastia, oligomenorrhea, amenorrhea, reduced libido, dyspareunia, vaginal dryness for women; reduced libido, erectile dysfunction, ejaculatory dysfunction for men (Gardner and Teehan, 2011; Li et al., 2014). Osteoporosis, hypogonadism, and breast cancer have been associated with prolactinomas, in which prolactin levels can exceed 5000 ng/ml (Gardner and Teehan, 2011; Li et al., 2014). It has been recommended that, prolactin should be measured at baseline and over the follow-up period (monitoring hyperprolactinemia-related signs and symptoms) (Ventriglio et al., 2014). Prolactin levels range from 0 to 20 ng/ml (0–424 mIU/l) in non-pregnant women: whereas levels for pregnant women are lower than 200 ng/ml (≤4240 mIU/l). For men the levels should range from: 0 to 15 ng/ml (0–318 mIU/l) (Kratz et al., 2004).

Hyperprolactinemia occurs more frequently in females than males, despite lower doses used in women. Return to normal prolactin levels occurs rapidly upon discontinuation of treatment (Halbreich et al., 2003; Gardner and Teehan, 2011).

Hyperprolactinemia associated with antipsychotic medication has been observed with effects from risperidone = paliperidone = amisulpride = haloperidol (at 74–91%) which is greater than that for olanzapine (2–20%) which is higher than that related to quetipaine (not significant increase) which is higher than that for aripiprazole = ziprasidone (not significant increase) (Halbreich et al., 2003; Gardner and Teehan, 2011).

When prolactin levels are high, but lower than 150 ng/ml, clinician must consider a switch from the current antipsychotic to another which is not associated with a significant increase in prolactin levels while taking into account the risk of rebound in psychotic symptoms. They may consider introducing a dopamine agonist like cabergoline, bromocriptin, pergolide. If prolactin levels are higher than 150 ng/ml, then advice from an endocrinologist may help along with neuro-imaging investigations to exclude pituitary and juxtasellar adenomas (De Hert et al., 2014).

## QT- interval prolongation

Using electro-cardiogram the functioning of the heart is assessed and in this context, QT interval is a measure of the time between the start of the Q wave and the end of the T wave in the heart's electrical cycle. In general, the QT interval represents electrical depolarization (Na^+^ and Ca^++^ intake in the cells) and repolarization (K^+^ output) of the ventricles. QT is corrected for heart rate (QTc) derived from the heart rate (HR) as 60 beats per minute according to Bazzett's formula.

Although QT may vary according to age, sex, circadian rhythms; a lengthened QT interval is a marker for the potential of ventricular tachyarrhythmias like *torsades de pointes* and a risk factor for sudden death. A QTc value above 500 ms or an increase of 60 ms (20% from baseline) is strongly associated with arrhythmias and torsade de pointes (TdP).

Antipsychotic agents may prolong QTc interval depending on their binding- potency to IKr channels of ventricle repolarization (HERG channels). Second generation antipsychotic mostly involved with QTc- prolongation are ziprasidone, risperidone, paliperidone, clozapine, quetiapine. aripiprazole, olanzapine, and asenapine are associate with lower variations in QTc- interval (Gardner and Teehan, 2011; Hasnain and Vieweg, 2014).

First generation antipsychotics are responsible for a higher QTc- prolongations, in particular drugs like haloperidol. Thioridazine, sertindole have consequently been withdrawn worldwide since these drugs lead to arrhythmias and sudden death. There is no doubt that good clinical practice demands that antipsychotic should be prescribed with caution for patients affected by cardiovascular diseases (congenital long QT syndrome, myocardial infarction, cardiac failure, arrhythmias) or other risky conditions (previous TdP or hypokalaemia) (Gardner and Teehan, 2011; Hasnain and Vieweg, 2014).

Finally, it has been reported that myocarditis may occur during the treatment with clozapine, in particular in the first 2 months of it (in up to 85% of cases). Also, patients treated with chlorpromazine or fluphenazine may develop myocarditis. If clinical signs of myocarditis are suspected (e.g., tachycardia), the drug should be stopped immediately (Gardner and Teehan, 2011).

## Monitoring metabolic disorders among patients affected by chronic psychosis

There have been many attempts by several international societies to develop guidelines for screening and monitoring metabolic consequences of psychosis and antipsychotic treatments. The aim of such procedures is to reduce those modifiable factors associated to the risk of cardiometabolic conditions among patients affected by schizophrenia (De Hert et al., 2009a). Guidelines developed by the European Psychiatric Association (EPA), supported by the European Association for the Study of Diabetes (EASD) and the European Society of Cardiology (ESC), also known as EPA/EASD/ESC Position Statement are very helpful in clinical settings (De Hert et al., 2009a; Table 2).

**Table 2:** **EPA/EASD/ESC Position Statement (adapted from De Hert et al., 2009a)**.

	**Baseline**	**AP Prescription**	**W 1**	**W 2**	**W 3**	**W 4**	**W5**	**W6**	**W12**	**M6**	**M9**	**1 year**
Weight	✓	✓	✓	✓	✓	✓	✓	✓	✓	✓	✓	
Height	✓											
BMI	✓	✓										✓
Waist circ.	✓	✓										
FBG	✓	✓						✓	✓			✓
TC	✓	✓						✓	✓			✓
TG	✓	✓						✓	✓			✓
LDL-C	✓	✓						✓	✓			✓
HDL-C	✓	✓						✓	✓			✓
HbA1c	✓	✓							✓	✓	✓	
Blood pressure	✓	✓										✓
HR	✓											
Chest auscul.	✓											
Pulse	✓											
ECG^§^	✓											
Other^*^	✓											✓

*Circ., circumference; auscul., auscultation. If patient is suffering from diabetes, HbA1c should be measured 3-monthly. Urinary albumin, creatinine, ophtalmology visit, and feet check- up should be planned annually*.

*BMI, body mass index; FBG, fasting blood glucose; TC, total cholesterol; TG, triglycerides; LDL-C, low density lipoprotein cholesterol; HDL-C, high density lipoprotein cholesterol; HbA1c, glycated hemoglobin; HR, heart rate; ECG, Electrocardiography*.

§ *ECG should be repeated yearly and in case of drug and dose changes*.

* *Any other medical assessment, if needed; W, week; M, month*.

For a number of reasons, these recommendations have been poorly followed and employed in the clinical settings. It is well known that when applied, these guidelines appear to improve the metabolic outcome of patients affected by schizophrenia and bipolar disorders (De Hert et al., 2009a, 2011; Ventriglio et al., 2014).

The baseline examination of patients should include history of previous cardiovascular diseases, diabetes or other related disease; family history of premature CVD, diabetes or other related disease; smoking habits, food selection, physical activity; weight and height in order to calculate body mass index (BMI) and waist circumference; fasting blood glucose levels; fasting blood lipids: total cholesterol, triglycerides, LDL cholesterol (by calculation) and HDL-C; blood pressure (measured twice and average taken), heart rate, heart and lung auscultation, foot pulses; ECG (De Hert et al., 2009a).

If metabolic parameters are within the normal range in patients on antipsychotic treatment for more than 12 months, it is recommended that clinicians must repeat previous factors annually. If values are in normal ranges at baseline in drug- naïve patients, monitoring has to be repeated at week 6, 12, and annually (paying particular attention to weight gain within the 6 week of treatment) (De Hert et al., 2009a).

In presence of one or more risk factors, the antipsychotic treatment must be selected carefully, matching the efficacy and safety (metabolic) perspective. Specific treatments may be required to reduce the cardiovascular risk. Clinicians have to involve general practitioners in order to collaborate with them in managing the risk factors. EPA guidelines recommend that BMI should be ≤25 kg/m^2^, waist circumference < 102 cm (men)/<88 cm (women), blood pressure ≤140/90 mmHg—triglycerides < 190 mg/dL—LDL < 115 mg/dL in non-diabetic patients. The, glycosylated hemoglobin < 7%—blood pressure ≤ 130/80 mmHg—triglycerides < 175 mg/dL—LDL < 100 mg/dL should be the recommended levels in patients with diabetes. Patients with diabetes should have their glycosylated hemoglobin levels measured 3-monthly and microalbuminuria, renal functioning, ophtalmological and feet examination yearly (De Hert et al., 2009a).

## Conclusions

Metabolic consequences of treatment with antipsychotic medication increase the risk of mortality among patients affected by chronic psychosis and many of the factors are preventable. Patients with psychoses appear to show higher levels of cardiometabolic co-morbidites in comparison with general population and 2–3-fold increased risk of premature death. Clinicians need to employ international guidelines and procedures to screen and monitor these co-morbid conditions in order to reduce mortality rates among patients affected by psychotic disorders and improve the outcome of illness, including patients' global functioning and quality of life.

## Funding

The research has been conducted in the absence of grant funding.

### Conflict of interest statement

The reviewer Marcello Nardini declares that, despite having collaborated on an article with authors Antonio Ventriglio and Antonello Bellomo in 2014, the review was conducted objectively and no conflict of interest exists. The authors declare that the research was conducted in the absence of any commercial or financial relationships that could be construed as a potential conflict of interest.

## Footnotes

^1^ADA Cardiometabolic Risk Initiative: http://professional.diabetes.org/resourcesforprofessionals.aspx?cid=60379 (accessed 02.08.2015).
